# The role of cholesterol-enriched diet and paraoxonase 1 inhibition in atherosclerosis progression

**DOI:** 10.15171/jcvtr.2017.23

**Published:** 2017-08-31

**Authors:** Masoumeh Amani, Akbar Darbin, Masoud Pezeshkian, Abbas Afrasiabi, Naser Safaie, Ahmadreza Jodati, Masoud Darabi, Maghsod Shaaker, Zeinab Latifi, Amir Fattahi, Gholam-Hossein Farjah, Mohammad Nouri, Mohammad Hassan Khadem-Ansari

**Affiliations:** ^1^Department of Clinical Biochemistry, Faculty of Medicine, Urmia University of Medical Sciences, Urmia, Iran; ^2^Department of Clinical Biochemistry, Faculty of Medicine, Tabriz University of Medical Sciences, Tabriz, Iran; ^3^Cardiovascular Research Center, Tabriz University of Medical Sciences, Tabriz, Iran; ^4^Department of Anatomy, Faculty of Medicine, Urmia University of Medical Sciences, Urmia, Iran

**Keywords:** Atherosclerosis, Atheroma, Paraoxonase 1, Nandrolone Decanoate

## Abstract

***Introduction:*** Atherosclerosis could be deemed as a chronic, progressive, and inflammatory disease. It has been well-documented that high-density lipoprotein (HDL) can reduce the risk of the atherosclerosis occurrence through exerting some anti-atherogenic mechanisms. In recent years, the strong evidence has suggested that paraoxonase 1 (PON1) may contribute to antioxidant properties of HDL. In the present study, the impact of a diet enriched with cholesterol and also the PON1 inhibition on atheroma formation and lipid profile has been investigated.

***Methods:*** In this study, 24 New Zealand rabbits were randomly assigned to three groups receiving standard diet, atherogenic diet, and atherogenic diet plus once daily intramuscular injection of nandrolone decanoate as the PON1 inhibitor. Triglyceride, cholesterol, HDL, and low-density lipoprotein (LDL) were determined and both cholesterol accumulation in aorta and fatty streak formation were evaluated.

***Results:*** The comparison of the results in three groups reveals that cholesterol level in the group received cholesterol-enriched diet plus once daily injection of PON1 inhibitor was higher than the groups received standard diet or atherogenic diet without PON1 inhibitor (*P *< 0.05). Furthermore, the percentage of atheroma with type-I lesions was equal to 75% compared with the group received atherogenic diet plus nandrolone at 30%. Additionally, the differences in fatty streak formation in aorta, as well as the right and left coronary arteries in three groups given show that the difference between groups receiving atherogenic diet and standard diet was significantly lower (*P *< 0.05) than the difference between groups receiving atherogenic diet plus PON1 inhibitor and standard diet.

***Conclusion:*** It can be concluded that lack of paraoxanase1 or even reduced the activity of this enzyme could accelerate the progression of fatty streak lesions toward advanced atherosclerotic lesions.

## Introduction

There are no reservations that cardiovascular diseases (CVDs) such as atherosclerosis underlie one of the leading causes of mortality in both developing and industrial communities.^1^ Atherosclerosis as a focal and chronic progressive inflammatory disease is characterized by the migration of monocytes/macrophages into the intima, pro-inflammatory cytokine secretion, cholesterol accumulation in the foam cells, and increased the intimal thickness.^2^ In terms of the mechanism involved in the formation of foam cells, uncontrolled uptake of oxidized- low-density lipoprotein (Ox-LDLs), impaired cholesterol efflux in macrophages and/or excessive cholesterol esterification lead to accumulation of esterified cholesterol stored as cytoplasmic lipid droplets. Subsequently, cholesterol accumulation causes the formation of foam cells in atherosclerotic lesions as the hallmark of early-stage atherosclerosis.^3,4^

As the atherosclerotic lesion progresses, intimal thickening is accompanied by accumulation of smooth muscle cells, T cells and apoB containing lipoproteins retention is amplified. While most of the fatty streak lesions are benign and found in even young individuals, intimal thickening may lead to clinically significant lesions by the formation of vulnerable plaques named ‘atheroma’.^5^

Evidence from both experimental and human studies have revealed the fact that HDL plays a vital role as the anti-atherogenic lipoprotein by reducing oxidative damage and inflammation, inhibiting the oxidation of LDLs, and also improving the endothelial function and macrophage-mediated cholesterol efflux. Furthermore, high-density lipoprotein (HDL) could decrease coronary atherosclerosis through decreasing the expression of adhesion molecules on endothelial cells which consequently leads to reduce the inflammation. Consequently, HDL paves the way for lipid peroxides and lysophospholipids to transfer from organs to the liver through hepatic scavenger receptors.^6^ Furthermore, perhaps, more importantly, HDL also could actually metabolize lipid hydroperoxides preventing their accumulation, and therefore, it can prevent the atherogenic structural modification of LDL.^7^ Several‏ lines‏ of‏ evidence‏ have‏ suggested that among enzymes associated with HDL, paraoxonase 1 (PON1) may contribute to antioxidant properties of HDL.^8,9^

PON1 as one of the HDL-associated enzyme in serum, is produced in the liver and protects LDL particles from oxidative modifications.^10,11^ It is believed that PON1 acts as a protein that is responsible for the most of the antioxidant properties of HDL.^7^ Owing that one of the initial steps for atherosclerosis is oxidation of LDL and production of Ox-LDL which can per se induce lipid accumulation and foam cell formation,^12^ and on the other hand due to pivotal role of PON1 in inhibiting LDL oxidation; it could be postulated that any change in activity of PON1 can affect LDL oxidation process and ox-LDL levels and consequently atherogenesis process. In support of this hypothesis many studies have reported association between functional polymorphisms of PON1 gene which could reduce the enzyme activity/levels and atherosclerosis.^13,14^ Besides some studies have reported association between low serum activity PON1 with CVDs^15^ and hypercholesterolemia.^16^ Up to now many factors have been introduced that can affect PON1 activity such as genetic, pathological, physiological, pharmacological and lifestyle.^17^ More interestingly animal studies have shown that PON1 activity can be modulated by diet.^18-20^ It is also reported that high–fat, high cholesterol (atherogenic) diet leads to the fast development of atherosclerosis in C57BL/6J mouse strain with a concomitant decrease in liver PON1 expression.^18^

However, although the current knowledge of PON1 provides valuable insights on the function and role of PON1, yet the role of this enzyme involved in the progression of atherosclerosis toward ‘atheroma’ is still not well investigated. Owing that involvement of PON1 as well as diet in atherogenesis has been revealed, in the present study we tried to investigate possible effect of PON1 in inhibiting of atheroma formation following atherogenic diet. For this purpose we used recently introduced anabolic steroid nandrolone which can cause a significant decrease in serum PON1 activity.^21^ So the impact of atherogenic diet (enriched with cholesterol) with and without nandrolone decanoate as the PON1 inhibitor, on lipid profile and their probable relation to atheroma formation in the aorta and coronary arteries has been studied in rabbit as an animal model.

## Materials and Methods

### Animal model

In this study, 24 New Zealand rabbits weighing 2 kg on average were obtained from Pasteur Institute of Iran (Tehran, Iran). The animals were kept under similar laboratory conditions (18°C to 23°C room temperature and controlled humidity) exposed to light–dark cycle of 12 hours, with free access to water and food. After a two-week acclimation period, the animals were randomly assigned to three groups. The group A was assigned, as the control group and received standard diets (pellets); group B (Atherogenic) received the same diet but supplemented with cholesterol; and group C (Atherogenic plus inhibitor) received both cholesterol-enriched diet and once daily intramuscular injections of nandrolone decanoate, as a PON1inhibitor. The administration period was eight weeks in all study groups.

To prepare the atherogenic diet, 2% Cholesterol were added to the standard diet. Briefly, to induce hypercholesterolemia 32 g of cholesterol powder ((Merck, Germany) added to water used for preparing the food and then, it was mixed with 1600 g normal rabbit food. The prescribed amount of atherogenic diet was 100 g per rabbit per 24 hours. At the end of 8 weeks, the animals were anesthetized with 5% sodium pentobarbital and euthanized under standard conditions and subsequently, samples taken from their vascular system were fixed in 10% formalin to evaluate atherosclerotic lesions including cholesterol accumulation and fatty streak lesions formation. Animal studies were performed in accordance with the ethics committee of Urmia University of Medical Sciences.

### Serum samples

At the first and end of 8 weeks, blood samples (5 mL) were collected for biochemical analyses. The sera were immediately separated by centrifugation at 1000 g (unit of gravity) for 5 minutes. Then, all aliquots were stored at -76°C until the analyses were carried out. In the all three groups and before and after treatment, triglyceride (TG), cholesterol, HDL, and LDL were determined using commercial enzymatic-colorimetric method (Pars-Azmoon, Tehran, Iran).

### Cholesterol accumulation in aorta

To measure the cholesterol deposits, a part of the aorta was dissected and then tunica adventitia was carefully removed using a dissecting microscope. In the next step, both the tunica media and the tunica intima were separated and added to tubes containing 3 mL of chloroform/methanol 2:1 (v/v) and cholestane as an internal standard, to extract and measure the lipids. Lipid extraction was separated from proteins by filtration. The samples were dried at 60ºC under a nitrogen stream and then, dissolved in hexane. The determination of free and total cholesterol was carried out by gas-liquid chromatography at 300ºC using a Buck Scientific model 610 gas chromatograph and two injections technique was used for assessment of analytes on a capillary column (0.25 mm × 0.2 μm × 50 m, TRB-Sterol).^22^

### Evaluation of fatty streak formation

For evaluation of fatty streak formation, the rabbits were anesthetized with 5% sodium pentobarbital and euthanized under standard conditions. Briefly, the heart and aorta were washed in phosphatase buffered saline and fixed in 10% buffered formalin for histopathologic examination. Hematoxylin and eosin stained, formalin-fixed, paraffin embedded sections of the dissected aorta and coronary arteries were examined for atherosclerosis (grades I–VI) by a certified pathologist in a blind fashion under a light microscope.^23^

### Statistical analyses

Data are shown as mean ± standard deviation (SD)‏. The normality of distributions was assessed by Kolmogorov-Smirnov test and the parametric test was used for statistical analyses. The differences between three groups‏ were examined by one-way analysis of variance (ANOVA). Moreover, Tukey post hoc test was used for pairwise comparisons. The differences in levels of different parameters before and after the diet were performed by a paired *t* test. All statistical analyses‏ were performed with the statistical software package SPSS-16.0 (SPSS Inc., Chicago, USA). A *P *< 0.05 was accepted as statistically significant.

## Results

In this study, New Zealand rabbits used as an animal model; categorized into three groups based on their diet received including standard diet, atherogenic diet, and atherogenic diet plus nandrolone as a PON1 inhibitor. Following the treatment of the animals for two months, blood sampling was carried out and then their heart and aorta tissues were investigated in terms of atherosclerosis progression.

Table 1 shows the lipid profiles in three groups given. The comparison of the results in these groups shows that the cholesterol levels are significantly different among three groups; the cholesterol levels in the group received cholesterol-enriched diet plus once daily injection of nandrolone was higher than the groups received standard diet or atherogenic diet only without PON1 inhibitor (*P *< 0.05). The cholesterol level, furthermore, in the group received cholesterol-enriched diet was significantly higher than the control group (*P *< 0.05). Moreover, as shown in Table 1, TG, HDL, and LDL levels in group C received atherogenic diet plus PON1 inhibitor, was higher than two other groups (*P *< 0.05) and these parameters in group B was also significantly higher than group received standard diet (*P *< 0.05).

**Table 1:** The comparison of lipid profile levels in sera from groups with standard diet as a control, atherogenic diet as a group B, and atherogenic plus PON1 inhibitor as a group C

	**Control (n=8)**	**Atherogenic diet (n=8)**	**Atherogenic diet + PON1 inhibitor (n=8)**
Cholesterol (mg/dL)	168±7	202±13^a^	246±21^a, b^
Triacylglycerol (mg/dL)	100±4	176±11^a^	286±39^a, b^
HDL (mg/dL)	33±2.8	23±3.8^a^	17±2.9^a, b^
LDL (mg/dL)	118±11	158±19^a^	171±18^a^

Abbreviations: HDL, High-density lipoprotein; LDL, low-density lipoprotein. Significant difference (*P* < 0.05) in comparison with ^a^ Control group, ^b^ Atherogenic diet group using one-way ANOVA test following Tukey post hoc test.

The comparison of free cholesterol and esterified cholesterol in the aorta (Table 2) indicates that free cholesterol levels in group C with PON1 inhibitor was significantly higher than two other groups (*P *< 0.05). Additionally, free cholesterol and esterified cholesterol in the group received cholesterol in their diet was higher than control rabbits (*P *< 0.05).

**Table 2:** The comparison of free cholesterol and esterified cholesterol in aorta from groups with, atherogenic diet as a group B, and atherogenic plus PON1 inhibitor as a group C

	**Control (n=8)**	**Atherogenic diet (n=8)**	**Atherogenic diet + PON1 inhibitor (n=8)**
Free Cholesterol (mg/g protein)	21±3.1	30±4.2^a^	42±6.5^a, b^
Cholesterol ester (mg/g protein)	8±1.4	34±4.8^a^	48±5.6^a, b^

Significant difference (*P* < 0.05) in comparison with ^a^ Control group, ^b^ Atherogenic diet group using one-way ANOVA test following Tukey post hoc test.

**Figure 1:**
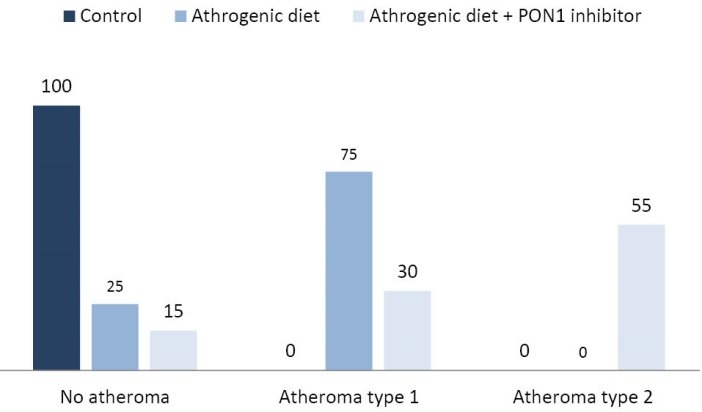
The comparison of atheroma formation percentage (with lesion type I and II) in three groups including groups with standard diet as a control, atherogenic diet as a group B, and atherogenic plus PON1 inhibitor as a group C.

As shown in Figure 1, The comparison of results shows that atheroma formation percentage in three groups using standard diet, cholesterol-enriched diet, and cholesterol-enriched diet plus PON1 inhibitor, the percentage of atheroma with type-I lesions was equal to 75% compared with the group received atherogenic diet plus nandrolone at 30%. In addition, the percentage of atheroma with type-II lesions in group B and C was 0% and 55%, respectively.

**Figure 2:**
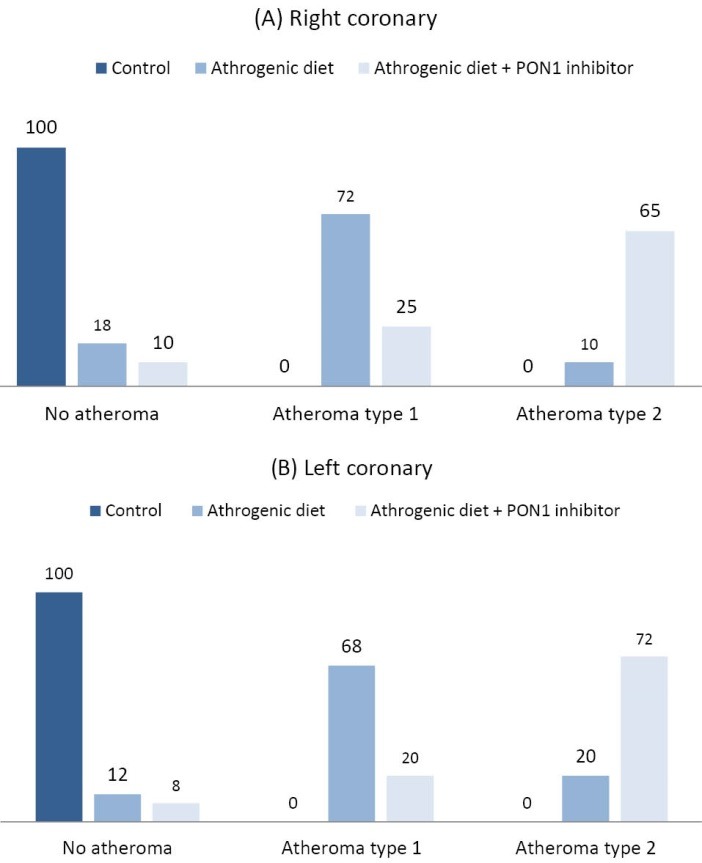
The comparison of atheroma formation in a. the right coronary arteries and b. the left coronary arteries in three groups including groups with standard diet as a control, atherogenic diet as a group B, and atherogenic plus PON1 inhibitor as a group C.

In our study, the atheroma formation percentage in coronary arteries of three groups of rabbits was also investigated indicating that type-I lesions percentage in the right coronary arteries of rabbits received cholesterol-enriched diet was at 72% compared with 25% in the group received atherogenic diet plus PON1 inhibitor (Figure 2A). Meanwhile, in the left coronary arteries, type-I lesions percentage in group B were at 68% compared to 20% in group C (Figure 2B). Furthermore, atheroma with type-II lesions in the right coronary arteries was formed at 10% and 65% of rabbits in group B and C, respectively (Figure 2َََََA) which was slightly lower than those of the left arteries stood at 20% and 72% of group B and C, respectively (Figure 2B).

The differences in fatty streak formation in aorta, as well as the right and left coronary arteries in three group provided in Table 3. In the case of aorta, our results indicated that the difference between groups receiving atherogenic diet and standard diet (2.4±0.5) was significantly lower (*P *< 0.05) than the difference between groups receiving atherogenic diet plus PON1 inhibitor and standard diet (3.0±0.6). Moreover, this difference between the groups receiving cholesterol-enriched diet and cholesterol-enriched diet plus the enzyme inhibitor (1.2±0.4) was significantly lower (*P *< 0.05) compared with the difference between groups received atherogenic diet and standard diet (2.4±0.5). This comparison was also drawn in coronary arteries and the results reveal that in the right coronary, the difference of fatty streak formation between the group B and C (0.6±0.2) was significantly lower (*P *< 0.05) than the difference between the group A and B (2.8±0.6). In the evaluation of fatty streak formation in the left coronary arteries, similar results also achieved (Table 3). Similarly, the difference between group receiving atherogenic diet and atherogenic diet plus nandrolone (0.7±0.2) was significantly lower (*P *< 0.05) than the difference between group control receiving standard diet and group C receiving atherogenic diet plus PON1 inhibitor (3.8±0.9) and also lower than difference between the animals in group control and group B (3.1±0.8).

**Table 3:** The differences in fatty streak formation in the aorta, the right coronary arteries, and the left coronary arteries of groups with standard diet as a control, atherogenic diet as a group B, and atherogenic plus PON1 inhibitor as a group C

	**Control VS. Atherogenic diet**	**Control VS. Atherogenic diet + PON1 inhibitor**	**Atherogenic diet VS. Atherogenic diet+ PON1 inhibitor**
Aorta	2.4±0.5	3.0±0.6^a^	1.2±0.4^a, b^
Right coronary	2.8±0.6	3.2±0.7	0.6±0.2^a, b^
Left coronary	3.1±0.8	3.8±0.9	0.7±0.2^a, b^

Significant difference (*P* < 0.05) in comparison with ^a^ Control group, ^b^ Atherogenic diet group using one-way ANOVA test following Tukey post hoc test.

## Discussion

There is strong in vitro and in vivo evidence that human serum paraoxonase (PON1) is closely associated with HDL and could prevent lipid peroxide accumulation on LDL.^7,8,24^ PON1 also could play a role in protecting macrophages against stress oxidative through hydrolysis of oxidized lipids, inhibition of LDL oxidation, and a decrease in the oxidized LDL uptake.^25^ Moreover, this enzyme could trigger the hydrolysis of esterified cholesterol and oxidation of some specific phospholipids.^26^ It has also been reported that there is an inverse relation between PON1 activity and the risk of CVDs. Therefore, PON1 level and activity could serve as an anticipating parameter for CVD.^13,27^ Moreover, the role of upregulated or downregulated PON1 gene in the development of atherosclerosis is also investigated in animal models.^28^ The finding of these studies implies that in PON1 knockout mouse, the atherosclerosis progression was accelerated compared with control mouse.^29^

In the present study, the role of a diet enriched with cholesterol and also atherogenic diet plus nandrolone as a PON1 inhibitor in the accumulation of cholesterol and atheroma lesions formation in the aorta and coronary arteries of rabbits have been studied. Our results proved that LDL, TG, and cholesterol levels in animals consumed atherogenic diet plus PON1 inhibitor, were significantly higher compared with two other groups (*P *< 0.05) which was concurred with other studies. Yang et al^30^ showed that cholesterol-enriched diet could increase the cholesterol levels and cause impaired vasodilation. In their study, it was confirmed that impact of atherogenic diet could be made on the function of the vessels rather than their structures, and subsequently, it causes the fatty streak formation in rabbits. Besides previously higher levels of serum total cholesterol and LDL-cholesterol in men carrying low activity allele of PON1 in compared with high activity allele carriers have been demonstrated^31^ which is in good accordance with our findings.

The comparison of free cholesterol levels and esterified cholesterol levels in three groups including control, receiving atherogenic diet, and receiving atherogenic diet plus once daily nandrolone injection indicated that free cholesterol level in the group receiving cholesterol-enriched diet plus enzyme inhibitor was significantly higher than two other groups (*P *< 0.05).

In our study, regarding the increase in fatty streak formation in the group receiving PON1 inhibitor, it can be inferred that PON1 could prevent the formation of foam cells in favor of limiting the formation of atheroma lesions. To confirm this hypothesis, the comparison of the percentage in terms of atheroma formation has been drawn among three group given and the results clearly showed that the percentage of atheroma with type-I lesions formed in group B receiving atherogenic diet was higher than those of group C receiving PON1 inhibitor. Moreover, the percentage of atheroma lesions in group B was significantly higher than group control. Unfortunately, we did not evaluate serum activity of PON1 which in addition of confirming the inhibitor effect, could give us opportunity to evaluate possible association between serum PON1 activity and atheroma lesions types. Finding such association could introduce serum PON1 activity as a possible biomarker for fatty streak and atheroma levels in aorta and coronaries.‏ Besides information about composition of fatty streaks could help to better understand underlying mechanism of fatty streak and atheroma formation following PON1 activity reduction. Future studies should take these points into the account to shed more light on mechanism of PON1 as an anti- atherogenic factor. Nevertheless, there are incontrovertible evidences agreed with the findings of the present study. In a study carried out by Takamoto et al^32^ on atheroma progression, it was demonstrated that the high levels of cholesterol lead to increase in esterified cholesterol modified by esterase which leads to a surge in its uptake by macrophages and subsequently accelerate the development of atheroma. Furthermore, it has been well-documented that amplified levels of cholesterol lead to increase in LDL in serum increasing the LDL oxidation. Concerning the fact that ox-LDL enhance the permeability of the arteries to LDL, hence, in intima of the aorta, increased amount of LDL and ox-LDL could trigger the accumulation of cholesterol and formation of fatty streak.^33^ Aviram et al^34^ reported that PON1 inhibition by ox-LDL could reduce the paraoxonase/arylesterase activity and then, leads to decrease protection against oxidation of LDL. Regarding the protective role of paraoxonase against atherosclerosis progression, therefore, it can be concluded that amplified ox-LDL could pave the way for atherosclerosis progression toward atheroma and CVD through PON1 inhibition. Navab et al^35^ also showed that reduced activity of PON1 causes declined ability of HDL against oxidation of LDL which can trigger the formation of atheroma plaques, atherosclerosis, and CVDs. Hence, in patients with CVDs, reduced activity of PON1 could be resulted from lower levels of HDL.^36^ Moreover, any intervention in order to increase PON1 activity could be considered as potential therapeutic approach for CAD patients. In support of this idea, it has been shown that in human PON1 transgenic mice with 2- to 4-fold higher activity of plasma PON1 and fed high fat diet, , the atherosclerotic lesions were significantly decreased.^28^ On the other hand, it has been revealed that plasma activity of PON1 could be affected by various environmental factors such as diet, smoking and alcohol consumption,^37^ so changing life style could has beneficial effects on cardiovascular conditions partly through increasing PON1 activity. Beside, future pharmacological studies about atherosclerosis could focus on producing medications with ability of inducing PON1 activity or expression. More interestingly there are controversial findings about effect of lipid lowering-drugs on PON1 activity and expression; as several studies have reported positive and some negative effects of statins on PON1.^37^

Moreover, it has been demonstrated that biological factors affecting the atheroma formation could make impacts on levels and activity of the enzyme.^38^ In a study by Chait et al,^39^ increased amount of the PON1 in the walls of the arteries with atheroma lesions has been described. However, the probable effects of this accumulation are not understood yet. Moreover, it has been reported that there is an inverse relation between PON1 levels and lipid peroxidation.^40^ Pezeshkian et al^41^ reported that paraoxonase inhibitor could accelerate the atheroma formation in rabbits. Thus future studies should pay more attention to mechanism of effect of PON1 on atheroma formation and establishment of novel therapeutic approaches for atherosclerosis prevention/treatment via manipulating PON1 availability and activity.

## Limitations of study

In the current study we did not evaluate serum activity of PON1 as well as composition of fatty streaks which possible could help to extract better conclusion.

## Conclusion

Simultaneous administration of cholesterol-enriched diet, as an atherogenic diet, and paraoxonase inhibitor cause significant changes in lipid profile, fatty streak formation, and also the levels of cholesterol and esterified cholesterol in aorta inducing atherosclerosis progression. Moreover, the formation of atheroma with type I lesions in rabbits receiving atherogenic diet and atheroma with type II lesions in the group receiving atherogenic diet plus PON1 inhibitor imply the role of the paraoxonase against atheroma formation. Therefore, it can be concluded that lack of this enzyme or even reduced the activity of the PON1, could accelerate atherosclerosis and CVDs.

## Competing interests

All authors declare no competing financial interests exist.

## Ethical approval

This study approved by the ethics committee of the Tabriz University of Medical Sciences.

## Acknowledgments

Authors appreciate the financial support granted by the department of Clinical Biochemistry, Faculty of Medicine, Urmia University of Medical Sciences. Moreover, the authors wish to thank the Vice Chancellor for research of Urmia University of Medical Sciences and Cardiovascular Research Center, Tabriz University of Medical Sciences.
